# N-terminal titin fragment: a non-invasive, pharmacodynamic biomarker for microdystrophin efficacy

**DOI:** 10.1186/s13395-023-00334-y

**Published:** 2024-01-16

**Authors:** Jessica F. Boehler, Kristy J. Brown, Valeria Ricotti, Carl A. Morris

**Affiliations:** 1 Solid Biosciences, 500 Rutherford Avenue 3rd Floor, Boston, MA 02129 USA; 2 Rejuvenate Bio, 11425 Sorrento Valley Road, San Diego, CA 92121 USA; 3 National Institute for Health and Care Research Great Ormond Street Hospital Biomedical Research Centre/University College London Great Ormond Street Institute of Child Health, London, UK; 4 PHDL Consulting LLC, 43 Sylvanus Wood Lane, Woburn, MA 01801 USA

## Abstract

**Background:**

Multiple clinical trials to assess the efficacy of AAV-directed gene transfer in participants with Duchenne muscular dystrophy (DMD) are ongoing. The success of these trials currently relies on standard functional outcome measures that may exhibit variability within and between participants, rendering their use as sole measures of drug efficacy challenging. Given this, supportive objective biomarkers may be useful in enhancing observed clinical results. Creatine kinase (CK) is traditionally used as a diagnostic biomarker of DMD, but its potential as a robust pharmacodynamic (PD) biomarker is difficult due to the wide variability seen within the same participant over time. Thus, there is a need for the discovery and validation of novel PD biomarkers to further support and bolster traditional outcome measures of efficacy in DMD.

**Method:**

Potential PD biomarkers in DMD participant urine were examined using a proteomic approach on the Somalogic platform. Findings were confirmed in both *mdx* mice and Golden Retriever muscular dystrophy (GRMD) dog plasma samples.

**Results:**

Changes in the N-terminal fragment of titin, a well-known, previously characterized biomarker of DMD, were correlated with the expression of microdystrophin protein in mice, dogs, and humans. Further, titin levels were sensitive to lower levels of expressed microdystrophin when compared to CK.

**Conclusion:**

The measurement of objective PD biomarkers such as titin may provide additional confidence in the assessment of the mechanism of action and efficacy in gene therapy clinical trials of DMD.

**Trial registration:**

ClinicalTrials.gov NCT03368742.

**Supplementary Information:**

The online version contains supplementary material available at 10.1186/s13395-023-00334-y.

## Introduction

Duchenne muscular dystrophy (DMD) is a devastating, severe myopathy that results in muscle wasting over time, leading to loss of ambulation and premature death. The cause of the disease is a loss of function mutation in the *DMD* gene, which encodes for the protein dystrophin that is essential for muscle health [1–3]. Dystrophin acts both as a membrane stabilizer to support proper muscle contractions [4–8] and as a signaling molecule that assists in various functions throughout the myofiber [9–21]. In its absence, the muscle membrane is damaged, signaling pathways are disrupted and muscle force is reduced. Over time, the skeletal muscle is replaced with fat and fibrotic tissue [22], underpinning the progressive nature of the disease.

A hallmark of muscle damage is elevated circulating proteins, usually of muscle origin, that have either been actively released or passively leaked from the muscle [23–39]. This suggests that under conditions of muscle damage, there are physical ruptures and/or altered signaling pathways in myofibers, resulting in a myopathic signature that can be observed in biofluids such as serum, plasma, or urine. A well-characterized marker of muscle damage is serum creatine kinase (CK), which is a widely used diagnostic marker for DMD, as well as other muscle diseases [40]. However, while elevated CK is a reliable biomarker of early-stage disease, it has been shown to decrease over time as a result of the progressive muscle loss associated with disease progression in DMD. Hence, the utility of CK and other muscle-specific proteins that are not typically found in circulation is more robust during earlier stages of the disease [23].

To prevent the accrual of additional muscle damage and subsequent muscle loss, several gene therapies, including Elevidys™ (delandistrogene moxeparvovec-rokl) suspension, which received FDA accelerated approval approved for use in 4–5-year-olds [41], have been designed to restore expression of a functional, albeit shortened, form of the dystrophin protein. Adeno-associated virus (AAV)-mediated gene transfer involves systemic administration of an AAV vector containing a transgene that expresses a mini- or micro-dystrophin that is delivered to muscles throughout the body. Ongoing clinical trials using different AAV capsids and microdystrophin construct designs have reported protein expression in muscle biopsies at an average of ~ 20–50% of normal dystrophin levels [42–44]. These differing AAV microdystrophin gene therapies have demonstrated functional efficacy in the *mdx* mouse model of DMD and in the Golden Retriever muscular dystrophy (GRMD) dog model [45–49]. However, the functional impact of these rationally designed proteins is currently being investigated as microdystrophins do not exist in nature. Further, the relationship between the functionality of a given quantity of microdystrophin as compared to normal, full-length dystrophin is currently unknown.

Functional outcome measures such as the North Star Ambulatory Assessment (NSAA), the 6-min walk test (6MWT), and the 4-stair climb are standard efficacy assessments incorporated into DMD clinical trial designs with the objective of determining if treatments result in clinically meaningful changes for participants. However, these standard outcome measures exhibit variability both within and between participants [50, 51]. Supportive, noninvasive pharmacodynamic (PD) biomarkers such as serum CK are potentially useful adjuncts to contextualize functional outcome measures, as they represent objective measurements that are downstream of microdystrophin’s direct mechanism of action of stabilizing the muscle membrane. Further, biomarkers within circulation provide a snapshot of muscle health throughout the body. However, CK is known to have wide intra- and inter-subject variability [52, 53], is thought to passively leak from the muscle, and decreases with age in DMD [53], limiting its usefulness as a PD biomarker of therapeutic efficacy, especially in older participants.

Previous studies have shown differences in several circulating proteins across multiple biofluids using various proteomic methods that seem to be driven by the lack of dystrophin in skeletal and cardiac muscles [23, 24, 26, 27, 33, 36, 54, 55]. Some of the more highly characterized markers have included titin [23, 34–37, 56], troponin [35, 38, 57–61], MMP9/TIMP1 [26, 31, 32, 62, 63], myomiRs [28–30, 64–66], MDH2 [23, 55], CA3 [24–27, 67–70], and MYOM3 [23, 24]. Out of these markers, urinary titin has recently been shown to act as a PD biomarker in *mdx* mice treated with exon-skipping drugs [34]. To explore the potential of these and additional noninvasive PD markers in DMD, proteomic screens were carried out using Somalogic’s SOMAscan platform. The screens were performed to measure circulating (serum/plasma) and/or cleared (urine) proteins in biofluids from AAV9-CK8-microdystrophin-treated *mdx* mice, GRMD dogs, and DMD participants. Titin, a marker previously associated with muscle damage in DMD [23, 36], was found to better correlate with changes in expressed microdystrophin protein and was more sensitive to changes in drug efficacy when compared to serum CK. Thus, titin may be a useful biomarker adjunct to microdystrophin expression measures in gene therapy clinical trials for DMD.

## Results

### SOMAscan identifies the N-terminal fragment of titin in DMD participant urine

Urine is an attractive biofluid as it is less invasive than serum/plasma collection and reduces the burden for clinical trial participation. As such, characterization of the urinary proteome in DMD participants, which has been previously described using mass spectrometry and antibody-based assays, was performed [36, 54]. The SOMAscan platform from Somalogic employs a non-biased method using its proprietary SOMAmer® aptamers to detect the presence of a large array of proteins (~ 7000) in a given sample [71–73]. Urine obtained from DMD participants and healthy age-matched controls from a previously characterized cohort [74] were used to quantify proteins found in the urine that may be altered by the expression of dystrophin—and potentially microdystrophin—in the muscle. The top ten upregulated and downregulated proteins (Table 1) were identified and significance was assessed using a 2-way ANOVA.

**Table 1:** Altered protein expression in human urine

	SeqId	SomaId	TargetFullName	UniProt	Healthy relative fluorescent units (average +/- SD)			DMD relative fluorescent units (average +/- SD)			Fold change (DMD/healthy)
*Upregulated with disease*	11,352–42	SL006679	Titin	Q8WZ42	623.7	+/-	604.4	46,605.9	+/-	30,350.3	74.7
	24,967–4	SL008346	Protocadherin-1	Q08174	69.4	+/-	24.5	1016.6	+/-	1049.2	14.7
	15,324–58	SL005628	Ferritin light chain	P02792	4359.3	+/-	2279.8	53,953.3	+/-	55,065.0	12.4
	5934–1	SL000420	Ferritin	P02794|P02792	4943.7	+/-	2603.2	54,607.3	+/-	53,308.2	11.0
	12,649–80	SL004917	Malate dehydrogenase, mitochondrial	P40926	318.0	+/-	615.5	3368.6	+/-	7239.0	10.6
	13,534–20	SL008132	Myomesin-2	P54296	76.1	+/-	15.2	738.3	+/-	963.5	9.7
	15,534–26	SL004917	Malate dehydrogenase, mitochondrial	P40926	171.4	+/-	239.2	1260.7	+/-	2549.2	7.4
	3042–7	SL000164	Myoglobin	P02144	250.9	+/-	127.6	1752.3	+/-	1724.5	7.0
	17,435–43	SL008038	Electron transfer flavoprotein subunit alpha, mitochondrial	P13804	274.0	+/-	92.1	1850.0	+/-	4174.9	6.8
	2942–50	SL000396	Cytochrome c	P99999	1101.8	+/-	759.7	6108.9	+/-	5064.5	5.5
*Downregulated with disease*	13,671–40	SL000401	Neutrophil elastase	P08246	20,227.6	+/-	35,004.5	1404.9	+/-	1240.7	-14.4
	6557–50	SL008774	Leucine-rich repeat-containing protein 15	Q8TF66	30,992.7	+/-	31,754.8	1970.7	+/-	3220.1	-15.7
	2796–62	SL000424	Fibrinogen	P02671|P02675|P02679	15,869.5	+/-	33,798.1	879.0	+/-	1009.3	-18.1
	3074–6	SL003309	Lipopolysaccharide-binding protein	P18428	2607.0	+/-	5621.3	130.4	+/-	108.4	-20.0
	2381–52	SL000319	Complement C5	P01031	2456.8	+/-	5737.2	113.2	+/-	19.9	-21.7
	10,620–21	SL000548	Beta-microseminoprotein	P08118	21,789.0	+/-	31,754.2	559.6	+/-	371.2	-38.9
	4330–4	SL000062	Prostate-specific antigen	P07288	21,034.7	+/-	32,186.5	56.0	+/-	13.4	-375.4
	8468–19	SL000062	Prostate-specific antigen	P07288	33,404.9	+/-	50,720.0	79.1	+/-	10.4	-422.2
	21,232–39	SL002764	Benign prostate-specific antigen	P07288	38,882.1	+/-	58,563.6	82.4	+/-	10.0	-472.0
	13,699–6	SL000062	Prostate-specific antigen	P07288	16,810.9	+/-	26,057.2	33.9	+/-	5.8	-495.9

**Fig. 1:**
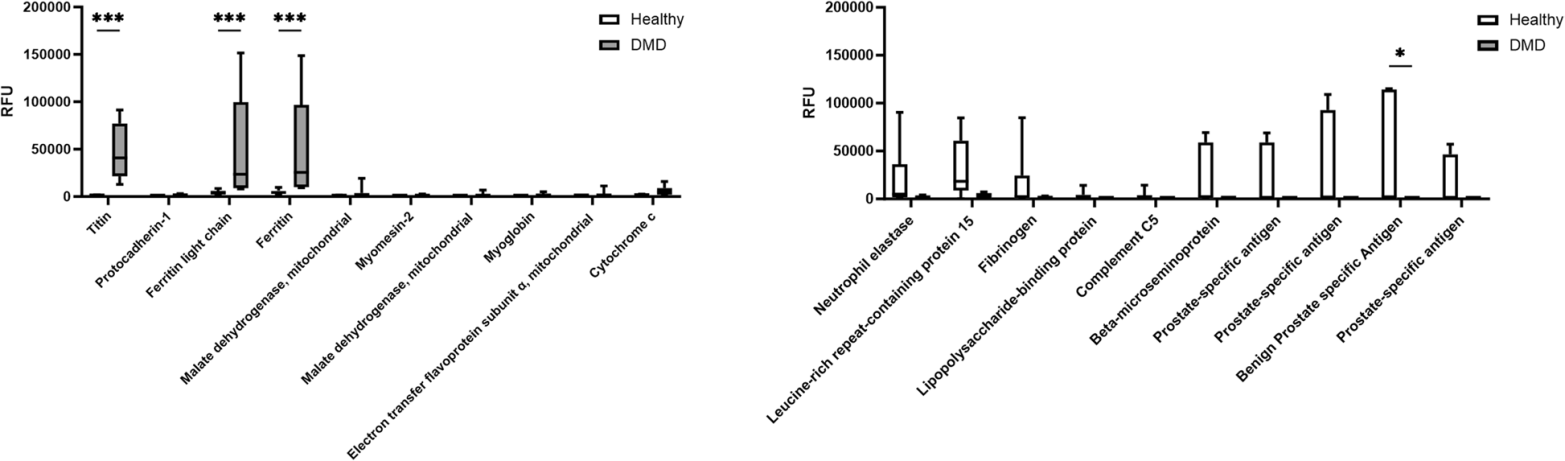
DMD urine proteome characterization using the Somalogic platform. SOMAscan assay identified (**a**) upregulated and (**b**) downregulated proteins in DMD urine when compared to healthy age-matched controls

Titin and ferritin proteins were found to be significantly upregulated (Fig. 1A), while benign prostate-specific antigen was significantly downregulated in DMD urine (Fig. 1B). Interestingly, increases in titin and ferritin have been previously described in DMD urine [36, 54], with titin being extensively characterized in the context of DMD disease biology [23, 34–37]. Due to the knowledge base already established for circulating and/or cleared titin’s role in DMD, titin was pursued as a potential PD biomarker in response to microdystrophin expression.

**Fig. 2:**
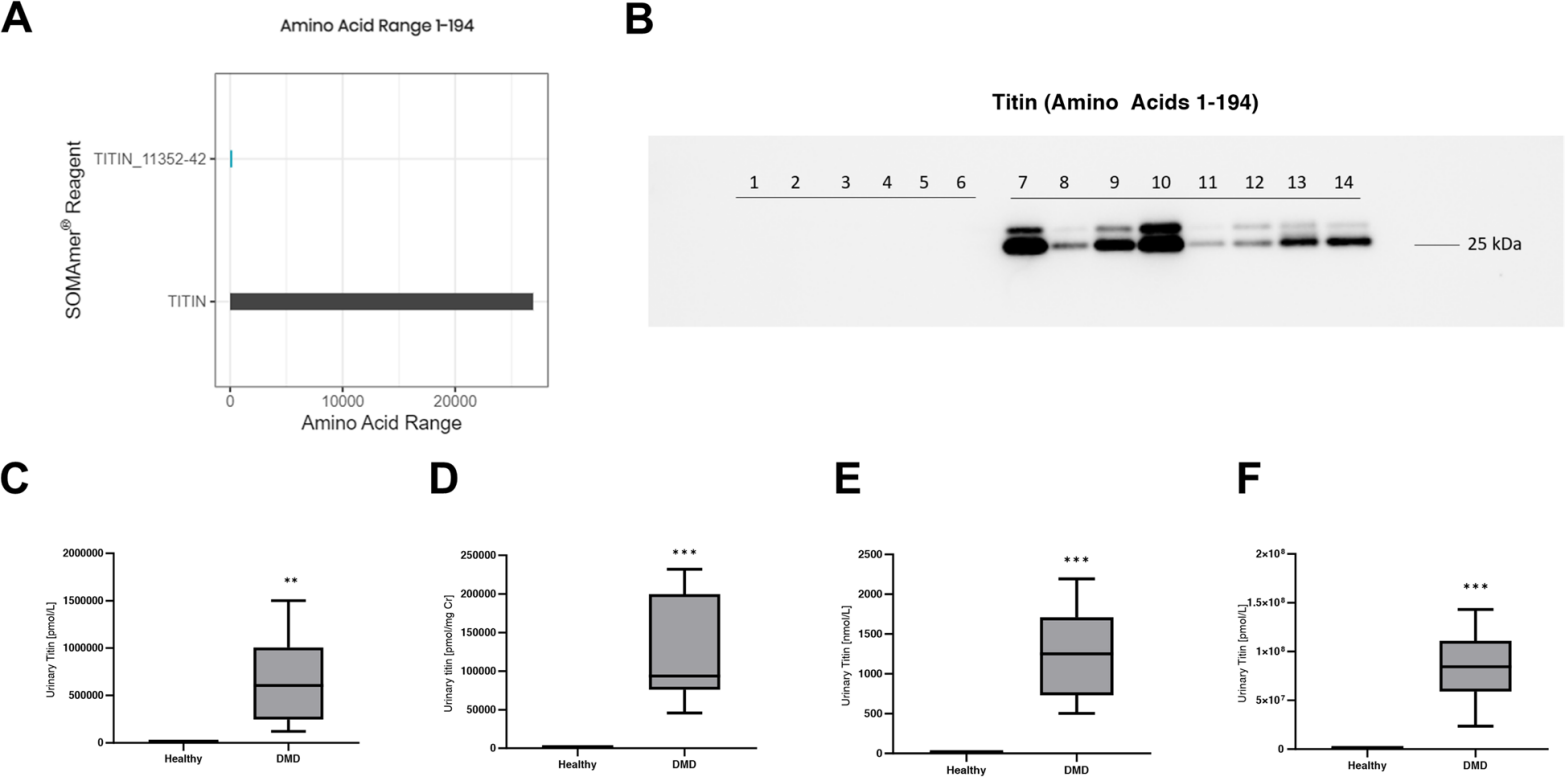
The SOMAmer detects the previously characterized N-terminal fragment of titin in DMD patient urine. **A** SOMAmer detects the N-terminal fragment (amino acids 1–194) of titin. **B** Western blot using anti-TTN mouse monoclonal antibody [clone: 7D3] against amino acids 1–110 shows the presence of the fragment in DMD (lanes 7–14) urine, as well its absence in age-matched healthy controls (lanes 1–6). **C** Human urine ELISA confirms increases in the titin fragment seen in the SOMAscan panel and Western blot. **D** Normalization of the ELISA results using creatinine, **E** specific gravity, and **F** cystatin C show significant increases in the urinary titin fragment in DMD urine. For individual plots of normalization values, see Supplementary Fig. 1

To gain additional confidence in the observed differences in titin quantities between DMD participants and healthy controls, Somalogic’s menu was searched and one SOMAmer aptamer that ostensibly detects the titin protein (SomaID SL006679) was identified. Somalogic has disclosed that this SOMAmer detects the N-terminal fragment of titin (amino acids 1–194) (Fig. 2A), which was expected since previous studies have identified this same fragment in DMD urine [36]. To confirm the SOMAmer result, a Western blot was performed using an antibody that detects the N-terminal fragment of titin. The titin protein fragment was only present in DMD urine (Fig. 2B), thereby replicating previously published data as well as confirming the SOMAmer results [36]. To quantify titin levels, a commercial ELISA developed against the N-terminal fragment was used to find a 267-fold increase in DMD participant urine compared to healthy age-matched controls (Fig. 2C).

Even though urine collections are easy and not limited by low volume amounts, it is a difficult biofluid to normalize when comparing across samples since many factors such as hydration and total volume collected can drastically impact results. To test different normalization factors, we used total creatinine (Fig. 2D), specific gravity (Fig. 2E), and cystatin C (Fig. 2F), all of which have been previously used in the context of DMD [34, 35, 37, 75] (Supplementary Fig. 1). Regardless of the normalization method, there was a strong association between elevated titin protein levels and DMD disease state.

### Urinary titin: a potential pharmacodynamic biomarker in DMD participants expressing microdystrophin

To test the applicability of titin as a potential pharmacodynamic biomarker in humans, urine samples were assessed using the SOMAscan assay at baseline, day 180, and day 360 from DMD participants treated with AAV9-CK8-μDys5 who participated in the IGNITE DMD clinical trial (NCT03368742). When samples were grouped by dose, we observed no statistically significant difference in urinary titin quantities; however, there was a wide range of microdystrophin expression in the muscle biopsies across participants.

Participants were segmented into one of two groups based on the percentage of microdystrophin-positive fibers from the vastus lateralis: Group 1 participants had < 10% microdystrophin-positive fibers and Group 2 had > 10% microdystrophin-positive fibers (Table 2).

**Table 2:** Clinical characteristics of patients run on somalogic urine panel

**Group**	**Microdystrophin expression cutoff based on %positive fibers by IF**	***n***	**Average age at dosing (years) +/- SD**	***p* value**	**Average baseline titin (RFU)** +/- **SD**	***p* value**	**Average baseline CK (U/L) **+/- **SD**	***p* value**	**Average %positive fibers by IF post day 90 administration**	***p* value**
1	< 10%	5	7.2 +/- 3.8	0.9590	36,106 +/- 41,196	0.8480	9131 +/- 4355	0.2556	2% +/- 4.47%	0.0045
2	> 10%	3	7.3 +/- 2.3		41,096 +/- / 10,172		12,876 +/- 3467		47% +/- 23.09%	

This grouping was justified based on previous literature showing that Becker muscular dystrophy, a milder and slower-progressing form of muscular dystrophy compared to DMD, is associated with > 10% of muscle fibers expressing a truncated form of dystrophin [76]. When comparing the groups, there were no differences with respect to age, baseline urinary titin, and serum CK activity levels. At day 90 post-dose, Group 1 participants had an average of 2% microdystrophin-positive muscle fibers, while Group 2 participants had an average of 47% microdystrophin-positive fibers.

**Fig. 3:**
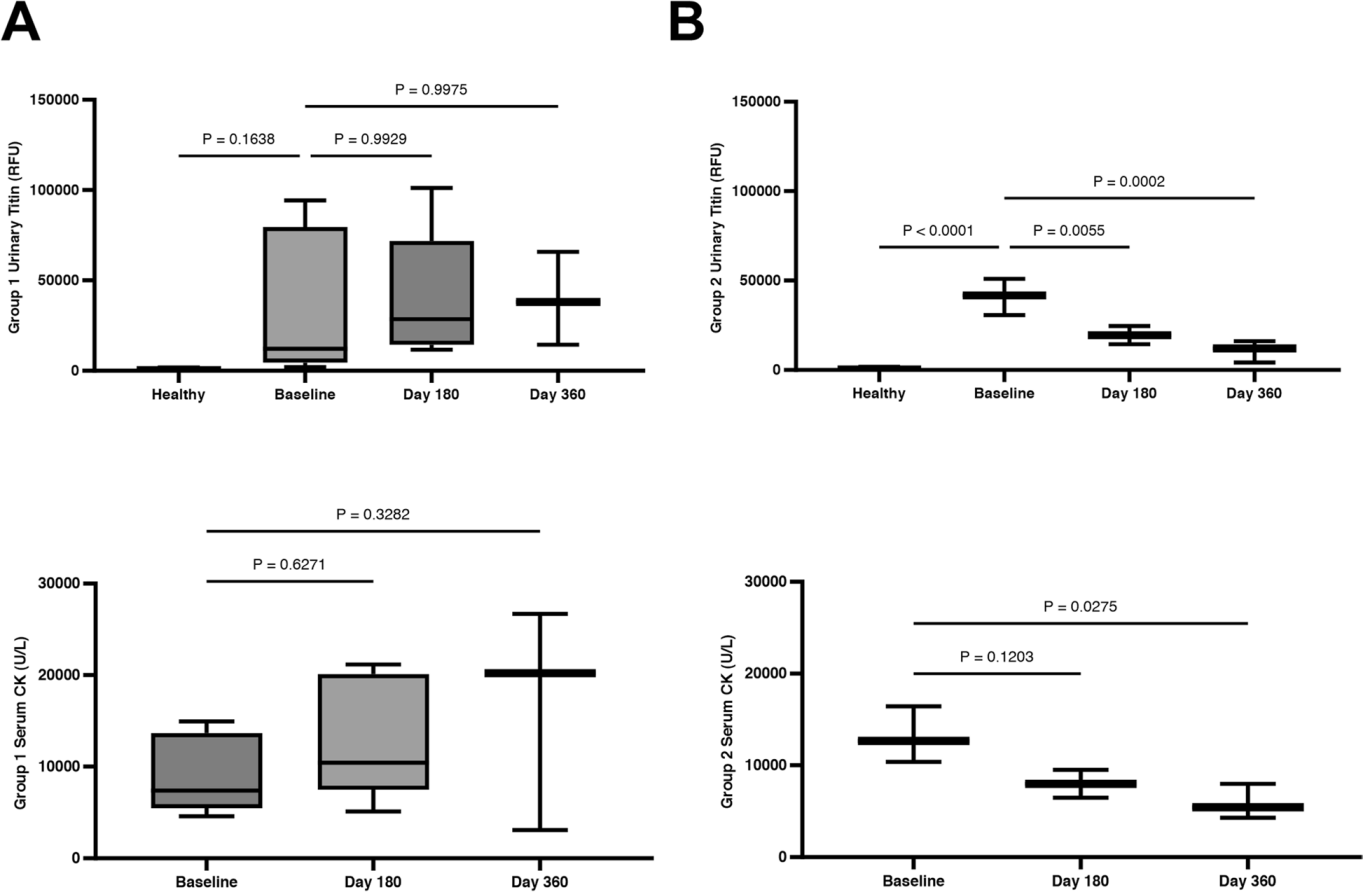
CK8-μDys5 reduces urinary titin in the presence of 40–50% microdystrophin-positive fibers. **A** Group 1 (microdystrophin < 10% positive fibers in the vastus lateralis) shows no changes in urinary titin or serum CK activity 360 days post-treatment. **B** Group 2 (microdystrophin > 10% positive fibers in the vastus lateralis) shows decreases in urinary titin at day 180 and 360, while serum CK activity was trending at day 180, but significantly changed at day 360 only

Urinary titin levels were quantified at baseline, day 180 and day 360 post-AAV9-CK8-μDys5 administration. Substantial, time-dependent decreases in urinary titin levels were observed in Group 2 participants, with both day 180 and 360 values reaching statistical significance compared to baseline levels, while the levels in Group 1 participants remained unchanged (Fig. 3). Similarly, serum CK activity showed a downward trend at day 180, but exhibited greater variability and did not reach a statistical significance until day 360 post-dose (Fig. 3).

### Circulating titin is highly conserved across multiple species and biofluids

**Fig. 4:**
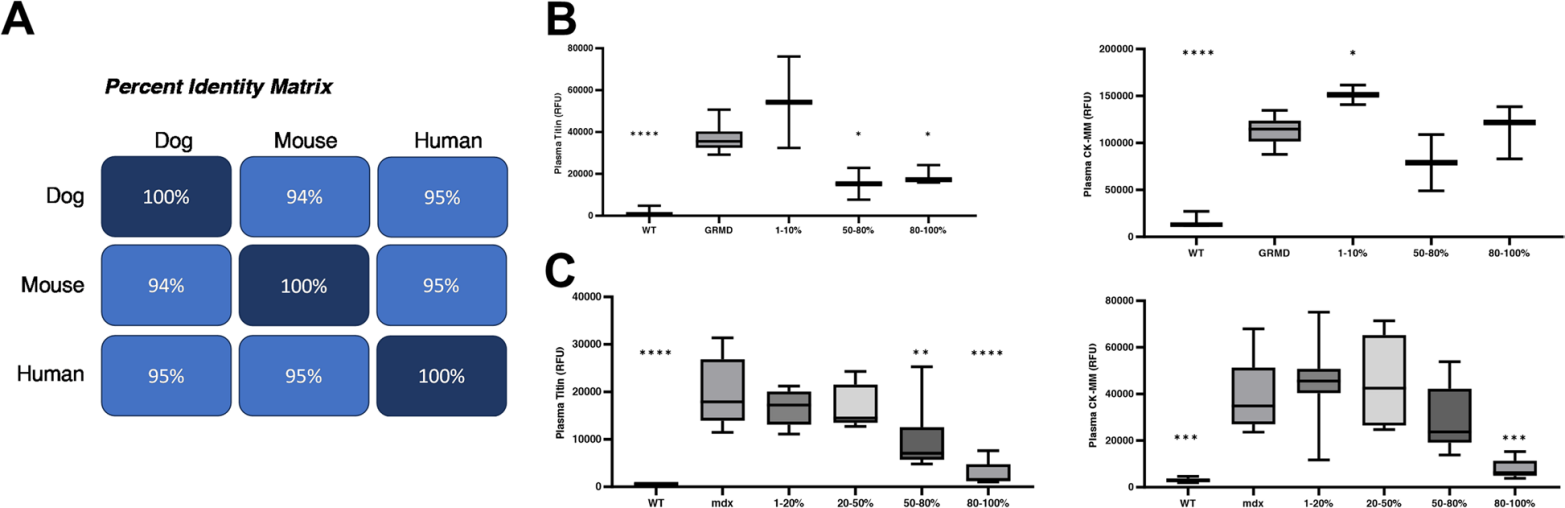
Circulating titin shows changes at lower levels of expressed microdystrophin when compared to CK-MM in preclinical DMD models. **A** The Somalogic aptamer detects the N terminal portion (AA 1–194) of the titin protein. This region was predicted to be highly conserved across mice, dogs, and humans. **B** Titin showed a response in vastus lateralis at > 50% microdystrophin levels, while an increase or no change was observed in CK-MM in the GRMD dogs 90 days post-treatment. **C** Circulating titin was also decreased in the plasma of *mdx* mice at lower levels of expressed microdystrophin in quadriceps when compared to CK-MM

Since the SOMAmer aptamer detects the N-terminal titin fragment, amino acid sequences across multiple species were compared to test if the Somalogic platform would be beneficial for testing preclinical samples. Uniprot sequence alignment of mouse, dog, and human to the aptamer found high sequence homology (95%) to humans when compared to both mouse and dog (Fig. 4A). Blood samples, not urine, were available from preclinical studies in *mdx* mouse and GRMD dog models; however, it was hypothesized that the N-terminal fragment may be present in blood as well due to the biomarker’s mechanism of action. As shown in Fig. 4, the SOMAmer was indeed able to detect the N-terminal fragment in plasma for both mouse and dog.

To model the clinical data and enhance confidence in our findings, the SOMAscan panel plasma from GRMD dogs that were treated intravenously with 1.0E13, 1.0E14, or 2.0E14 vg/kg AAV9-CK8-μDys5 was tested. The characterization of this cohort of GRMD dogs has been extensively described [46]. Plasma was assayed at 90 days post-treatment to replicate biopsy timepoints in which multiple clinical trials have reported microdystrophin expression data. The results showed a decrease in circulating titin in the range of 50–80% fibers positive for microdystrophin expression (≥ 1.0E14 vg/kg) in the vastus lateralis, while no changes in circulating CK-MM protein was observed at any dose or microdystrophin expression level (Fig. 4B). A similar trend was observed in *mdx* mice with decreases in circulating titin observed at significantly lower levels of microdystrophin (quantified by mass spectrometry in quadriceps) compared to CK-MM protein (Fig. 4C).

## Discussion

The standard functional outcome measures employed in clinical trials of DMD participants could benefit from the incorporation of additional supportive and objective endpoints for the assessment of therapeutic efficacy. Heterogeneity of disease phenotype, small participant numbers, and lack of sensitivity, as well as potential for subjective bias leave significant room for improvement in the clinical deployment and contextualization of these standard measures in a trial setting [77]. Measures must be compared to natural history to rule out changes due to disease; however, real-world evidence using characterized cohorts has limitations [78]. Placebo arms may impart more rigor to these assessments, but there are also significant ethical considerations around the use of placebo controls for participants with a rare, progressive, and universally fatal disease. Traditionally, functional measurements such as the NSAA, 6MWT, and 4-stair climb are used as definitive readouts to assess drug efficacy, but the sensitivity needed to detect potential changes in drug effects in a reasonable period of time is very high, especially when changes owing to disease progression occur over many years. Additionally, motivation is known to affect some outcome measures [79], so motivational bias cannot be ruled out when changes in the functional measurements are observed.

One way to bolster and substantiate current tools is the incorporation of objective biomarkers into clinical trial designs. Objective biomarkers are easier to identify in genetic diseases with a known defect. In DMD, microdystrophin expression is presumed to be a surrogate biomarker of clinical benefit based on extensive preclinical data in combination with known mechanisms of action and outcomes in participants with mutations that result in shortened dystrophin proteins [45, 46, 80]. Many have attempted to utilize serum CK as a biomarker to detect drug-induced effects in the context of clinical trials of DMD, but only trends have been observed thus far [81]. In addition, as this biomarker is known to decrease with increasing muscle loss during disease progression, it is not ideal for assessing drug efficacy over time, especially in older participants. To look for better candidate pharmacodynamic markers, urine samples from DMD participants who received AAV9-CK8-μDys5 were evaluated and changes in the N-terminal titin fragment were observed. By grouping participants according to microdystrophin expression levels, the effect of AAV transduction was controlled and showed that the decrease in the N-terminal fragment of titin appears to be driven by the presence of increased expression of microdystrophin protein in muscle fibers. Further, changes in titin were detected earlier post-treatment than changes in serum CK. This result was replicated in both the *mdx* mouse and the GRMD dog model, where alterations in plasma circulating titin occurred at lower levels of restored microdystrophin when compared to CK-MM.

Although the reason for the enhanced sensitivity of titin as compared to CK is currently unknown, a hypothesis is that the aptamer may exhibit greater sensitivity and/or specificity for the detection of titin vs. available reagents for quantification of CK-MM. The fact that this sensitivity was still present when compared to the CK activity assay in the IGNITE DMD trial suggests that additional biology could also be at play. It is known that titin has a slower rate of decline over time when compared to serum CK, so the window needed to detect a change could be greater, allowing for more sensitivity [55]. Additionally, it is also hypothesized that the N-terminal fragment has an active, specific proteolysis event that releases it into circulation, while serum CK is thought to leak passively [35–37]. More studies to analyze the overlapping and distinct biology around these markers would be beneficial.

While the added sensitivity of titin brings potential advantages towards evaluating therapeutic efficacy, since it is also a muscle-specific protein, similar to CK, it is also likely to decrease with age and the corresponding muscle loss in DMD [23, 37]. The optimal marker would possess a directional trajectory that is opposite that of the natural history of the disease, but this may be difficult to achieve as most muscle-specific proteins identified to date in biofluids increase early in the disease state and decline as muscle is lost. Therefore, additional objective biomarkers that remain stable through later stages of disease that may be paired with serum CK and the N-terminal titin fragment would increase confidence in drug effects. Regardless, additional studies of urinary titin through the natural history of DMD, as well as its performance over time in clinical trials and its ability to bolster the interpretation of both functional outcome measures and microdystrophin expression itself in therapeutic trials, may improve the ability to detect and quantify the benefits of therapeutic approaches aimed at restoring muscle integrity, such as microdystrophin gene therapy.

## Methods

### Study approval

The mouse study was performed according to the Dalhousie University Committee on Laboratory Animals under an approved protocol and in compliance with the *Canadian Council on Animal Care* guidelines at Agada Biosciences*.* The approval for the dog study was previously published [46] and all legal guardians and/or participants participating in the IGNITE DMD trial (ClinicalTrials.gov NCT03368742) provided written informed consent before enrollment.

### Statistics

All statistical tests were run in GraphPad Prism. Means and standard deviations were calculated for each group. For analysis that included more than two groups, an ordinary one-way ANOVA using multiple comparisons was selected and the mean of each group was compared with the mean of the vehicle-treated or baseline control. For analysis that included two groups, an unpaired parametric *t* test was used to identify significant changes in biomarkers.

### Biofluid collection

#### Plasma/serum

The blood was collected into K2EDTA tubes for plasma or 1.5 mL Eppendorf tubes for serum and immediately placed on ice. Samples were centrifuged for 10 min at 10,000 rpm at 4℃. Supernatants from each tube were aliquoted to two Eppendorf tubes and stored at − 80℃ until further analysis. For serum CK activity, quantification was determined using the Pointe Scientific Liquid Creatine Kinase Reagent Set (ref:C7522-450).

#### Urine

Urine was centrifuged at 1500xg for 10 min at 4℃ to remove cell debris, aliquoted, and stored at − 80℃ until assayed.

### Protein quantification

#### SOMAscan assay

Frozen plasma or urine aliquots were shipped to Somalogic, and mouse and dog plasma were run on SOMAscan 4 K, while the human urine was run on the SOMAscan Discovery Assay. Data were delivered in an ADAT file that contained normalized RFUs from their analysis pipeline.

#### Immunofluorescence

Isopentane frozen muscles were sectioned (8 microns) and stained for microdystrophin (MANEX44A, Developmental Studies Hybridoma Bank, University of Iowa), as previously described [46].

#### Mass spectrometry for mdx microdystrophin quantification

Dystrophin quantification was performed using methods previously published [46, 82, 83]. Briefly, in-gel digestion was performed on protein extracts from quadricep tissue spiked with a stable isotope internal standard. The resulting peptides were subjected to time-targeted parallel reaction monitoring nano-LC–MS/MS. Quantified microdystrophin protein was reported as a percentage of normal dystrophin calculated using a regression slope of a 5-point standard curve derived from a combination of protein from wild-type and dystrophin-deficient canine tissue.

#### Urinary titin

For Western blot analysis**,** 3μL of urine was separated by SDS-PAGE electrophoresis using a previously published protocol [36]. The N-terminal fragment was detected with titin (7D3) antibody (Novus Biologicals). For confirmation using ELISA, urinary titin levels were quantified with the IBL ELISA kit following the manufacturer’s instructions. For normalization, the creatinine colorimetric assay (Caymen Chemicals), specific gravity as measured by refractometer (Laxco Benchtop Digital), and cystatin C ELISA (Abcam) were used following the manufacturer’s protocol.

### Supplementary Information

**Additional file 1.**

## Data Availability

Not applicable.
