# Supplementary figures and images for: N-terminal titin fragment: a non-invasive, pharmacodynamic biomarker for microdystrophin efficacy
Source: Skelet Muscle. 2024 Jan 16;14:2. doi: 10.1186/s13395-023-00334-y (PMC10790446; doi:10.1186/s13395-023-00334-y)

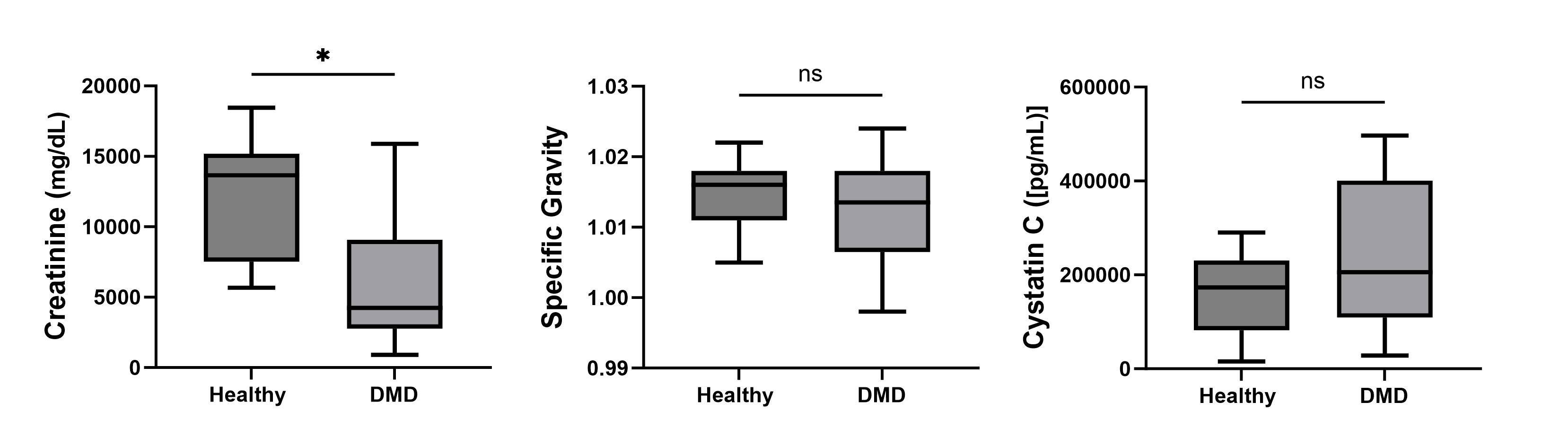

Supplement: Supplementary file 1 — Additional file 1. [file 13395_2023_334_MOESM1_ESM.tif]
